# Evaluation of Postharvest Senescence of Broccoli via Hyperspectral Imaging

**DOI:** 10.34133/2022/9761095

**Published:** 2022-05-09

**Authors:** Xiaolei Guo, Yogesh K. Ahlawat, Tie Liu, Alina Zare

**Affiliations:** ^1^University of Florida, Department of Electrical and Computer Engineering, Gainesville, Florida, USA; ^2^University of Florida, Horticultural Sciences Department, Gainesville, Florida, USA

## Abstract

Fresh fruit and vegetables are invaluable for human health; however, their quality often deteriorates before reaching consumers due to ongoing biochemical processes and compositional changes. We currently lack any objective indices which indicate the freshness of fruit or vegetables resulting in limited capacity to improve product quality eventually leading to food loss and waste. In this conducted study, we hypothesized that certain proteins and compounds, such as glucosinolates, could be used as one potential indicator to monitor the freshness of broccoli following harvest. To support our study, glucosinolate contents in broccoli based on HPLC measurement and transcript expression of glucosinolate biosynthetic genes in response to postharvest stresses were evaluated. We found that the glucosinolate biosynthetic pathway coincided with the progression of senescence in postharvest broccoli during storage. Additionally, we applied machine learning-based hyperspectral image (HSI) analysis, unmixing, and subpixel target detection approaches to evaluate glucosinolate level to detect postharvest senescence in broccoli. This study provides an accessible approach to precisely estimate freshness in broccoli through machine learning-based hyperspectral image analysis. Such a tool would further allow significant advancement in postharvest logistics and bolster the availability of high-quality, nutritious fresh produce.

## 1. Introduction

Broccoli (*Brassica oleracea* L. var. *italica*) is a nutritious vegetable that is also enriched in chemical compounds like glucosinolates that can lower cancer risks [1]. Glucosinolates function not only in plant protection, but also can reduce the risks for certain cancers in those who consume plants high in glucosinolates. Broccoli is usually harvested while the inflorescence is still developing and is removed from water and nutritional sources, which causes stress-induced senescence. This senescence leads to faster chlorosis and increases in protease activities that dismantle chloroplasts and cause chlorophyll breakdown [2].

Senescence is a developmental process accompanied by physiological and biochemical changes in transcripts, proteins, and metabolites at discrete stages. One particularly interesting class of metabolites are glucosinolates. Glucosinolates are present as glucosides in the brassicas and are decomposed inside the plant cells during cutting, damage, or chopping by the enzyme called myrosinase into smaller sulphur containing compounds such as isothiocyanates [3].

The glucosinolate content in brassicas depends on numerous factors, such as cultivar, harvest time, and storage conditions. Furthermore, both pre- and postharvest factors affect glucosinolate content in broccoli [2, 4]. It was reported recently that harvest time is crucial for the evaluation of glucosinolate contents in broccoli and harvesting at noon maintains higher level of glucosinolates [4]. In harvested broccoli, the actual quality, storability, and overall freshness are quite uncertain unless changes such as chlorophyll and carotenoid degradation are visible to human eyes [5].

Chlorophyll fluorescence and RGB (red, green, and blue) color imaging analyses were used to monitor pigment changes in broccoli during postharvest storage [6]. However, objectively measuring the progression during the loss of freshness in produce after harvest has always been an intractable problem in postharvest handling of fruit and vegetables. A strategy to measure the glucosinolate accumulation would detect any early physiological and biochemical changes of senescence before any visible signs occur that would allow for the early determination of freshness thus development of diagnostic tools for improvement of postharvest shelf life [7]. Therefore, sensors for initiation and progression of deterioration are essential for monitoring the physiological changes during postharvest storage in broccoli as well as other fruit and vegetables.

The rapid advancement of optical sensors such as multispectral imaging technologies has significantly impacted agriculture [8]. HSI provides both spatial and spectral information about an object and consists of thousands of pixels in a two-dimensional array, with each pixel containing a spectrum corresponding to a specific region on the surface of the sample. These spectra vary according to the material and chemical compositions. Introduction of these spectra provides the development of mathematical models to estimate chemical compositions or functional class of a sample associated with each pixel. HSI has been used in plethora of applications in the study of brassicas, to detect and quantify components and quality parameters in a wide range of biological matrices. For instance, recent studies estimated canola seed maturity using a UAV-mounted hyperspectral camera [9], quantified the oil content and fatty acid in Brassica seeds [10], and detected the nitrogen concentration in oilseed rape leaf with a laboratory hyperspectral imaging system [11].

Numerous techniques have been proposed to correlate hyperspectral data with fruit and vegetable quality and safety evaluations, including feature extraction via principal component analysis (PCA) [12, 13], linear discriminant analysis (LDA) [13, 14], partial least squares regression (PLSR) [14–17], classification via support vector machines (SVM) [15], random forests (RF) [16], and artificial neural networks (ANN) [15, 18] and recent work on spectral unmixing techniques [19].

To meet the need for detection of senescence progression through imaging sensors such as HSI, we leveraged HSI, applied spectral unmixing, and target detection algorithms to predict senescence at an early stage of symptoms. Compared with the previous works in dimensionality reduction and multivariate classification, our main contributions are as follows: (i) the application of spectral unmixing for physically meaningful and interpretable feature extraction; (ii) we estimated a spectral signature across all wavelengths for broccoli tissue with different glucosinolates concentration; and (iii) we generated improved maps which visualize the glucosinolates changes in broccoli. Here, we propose the accumulation of glucosinolates that correlated with onset and progression of senescence in broccoli following harvest. We first conducted biochemical measurements and gene expression analysis to illustrate that glucosinolates can serve as a freshness indicator before noticeable color changes in broccoli. Then, we demonstrated the feasibility of the HSI to determine the glucosinolate concentration of broccoli during postharvest storage. We concluded that a combination of glucosinolate analysis and HSI can evaluate the freshness signatures of postharvest broccoli.

## 2. Materials and Methods

### 2.1. Tissue Collection and Preparation

The crowns of freshly grown broccoli (cultivar, Emerald Crown) were manually harvested from local farms in Hastings, Florida. All broccoli florets were selected with similar size and small beads in this study. The florets were subjected to different storage conditions: 4 °C for cold treatment and 25 °C for room temperature. Four biological replicates of broccoli florets were sampled for tissue collection. Tissue samples were collected from the broccoli florets every other day over a twelve-day period. The samples were wrapped in aluminum foil, immediately frozen in liquid nitrogen, and then either stored at -80 °C for later quantitative PCR or freeze-dried for HPLC. The similar broccoli samples were chosen for the spectral imaging analysis.

### 2.2. Extraction of Total Glucosinolates for HPLC Quantification

HPLC analysis was performed to quantify the glucosinolates. Total glucosinolates were measured according to previously reported methods, with some modifications ([20]. Briefly, raw materials from harvested broccoli florets that were stored at -80 °C were taken out to be dispersed in liquid nitrogen. Broccoli tissue samples were weighed, and 100 mg fresh tissue was crushed to a fine powder using a mortar and a pestle. The tissue powder was dissolved in 1 ml of 50% methanol in a 1.5-ml microcentrifuge tube. The tubes were kept at 65 °C for 1 hour in a water bath. Samples were then centrifuged at 15000×*g* for 10 min. The supernatant was filtered through a 0.22 *μ*m pore size hydrophilic PTFE syringe filter (Sigma Aldrich, USA). The glucosinolate contents were estimated based on the peak area at 220 nm [21]. Four biological replicates were used for each time point.

### 2.3. RNA Extraction and Quantitative Real-Time PCR

Floret tissue samples from days 1, 3, and 5 were chosen for expression analysis of genes related to the glucosinolate biosynthetic pathway. Total RNA was isolated from broccoli floret using the TRIZOL (Ambion, Life Technologies, USA) method with DNase treatment (Turbo DNA free, Thermofisher, USA). First-strand cDNA synthesis from 1 *μ*g of total RNA was performed using a reverse transcription kit (Applied Biosystems, Foster City, CA). For the quantitative real-time RT-PCR, the primers were designed using Primer-Quest from Integrated DNA Technologies (IDT). The primers for the genes of glucosinolate biosynthetic pathway are listed in Table 1. Real-time PCR reaction was performed in an Applied Biosystems qPCR machine (Thermofisher, USA). The total reaction volume was 10 *μ*l for each gene, and the reactions were run in triplicate with thermocycler conditions as follows: 95 °C for 10 min, 45 cycles of 95 °C for 30 sec, and 60 °C for 30 sec. The relative gene expression was calculated by *ΔΔ*CT method according to the qPCR machine manufacturer (Thermofisher, USA). The *Actin2* from broccoli was used as an internal control. The experiment was repeated twice for expression studies.

### 2.4. Hyperspectral Image Acquisition

Broccoli hyperspectral images were collected with a HinaLea Model 4200 hyperspectral camera (TruTag Technologies, Emeryville, CA). The acquired raw images were calibrated to relative values with the white and dark reference:

(1) $$\begin{array}{c} \text{Calibrated}=\frac{\text{Raw}-\text{Dark}}{\text{White}-\text{Dark}}, \end{array}$$

and then converted to reflectance spectra with TruScope Software (TruTag Technologies, Emeryville, CA). The camera covered the visible wavelengths ranging from 400 nm to 700 nm (corresponding to chlorophyll absorption and sensitive development), the red-edge (chlorophyll content and stress conditions), and the near-infrared region (700 nm-1000 nm, related to cell structure) with a resolution at 4 nm (based on full-width at half maximum), resulting in 300 wavebands. 70 W halogen lamps (Malvern Panalytical Ltd, Malvern, UK) were used as illumination in the imaging chamber. Within the chamber, each broccoli sample was placed on a black plate with matte black sides that absorbs redundant light to minimize scattering. Hyperspectral measurements of four biological replicates were carried out at days 1, 3, 5, 8, 10, and 12 over the twelve-day period. The florets were imaged from the side where the lower part of the broccoli crown is near to the pedicle. The similar area of tissues were collected for glucosinolates analysis.

### 2.5. Hyperspectral Image Analysis

Considering that the HSI can detect changes in chemical compositions under various abiotic stress conditions, we reasoned that the initiation and progression of glucosinolates accumulation during postharvest senescence can be monitored through HSI analysis. To achieve this goal, we performed time course imaging experiments on broccoli florets under room temperature and refrigerated storage conditions.

**Figure 1:**
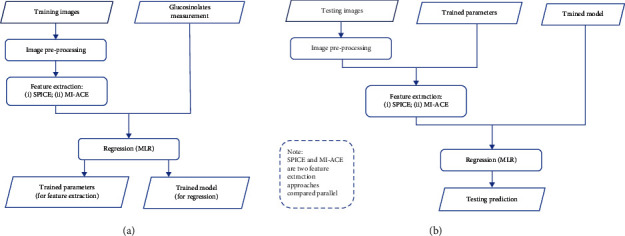
Flowchart of HSI data analysis. (a) Training procedure. (b) Testing procedure.

An overview of the analysis is shown in Figure 1, including training and testing procedures. Both training and testing phases started with several preprocessing steps. The goal of training was to estimate parameters for feature extraction and fit model for regression. Here, we compared two feature extraction approaches based on (i) spectral unmixing using the Sparsity Promoting Iterated Constrained Endmember (SPICE) [22] and (ii) subpixel target detection using the Multiple Instance Adaptive Cosine Estimator (MI-ACE) [23, 24]. Using each of these feature extraction approaches, the extracted features from each broccoli image was paired with a corresponding glucosinolates measurement and used to fit a multivariable linear regression (MLR) model. The trained parameters and trained model were then fed into a testing phase to predict the glucosinolate concentration of testing samples that were not used during training. The combination of feature extraction and regression procedure was compared to the classical PLSR approach. An overview of this approach is shown in Figure 1.

### 2.6. Hyperspectral Image Preprocessing

**Figure 2:**
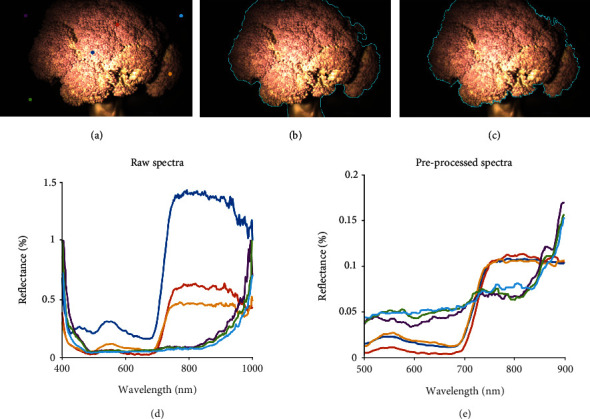
RGB imaging segmentation procedure and processed spectra. (a) Broccoli sample placed on a black plate. (b) Segmented broccoli head. (c) Segmented broccoli crown without stem. (d) Reflectance measured by HinaLea 4200 hyperspectral camera. (e) Preprocessed spectra. The colors of spectra in (d-e) correspond to the colored points in (a).

For this study, preprocessing of the measured spectra for noise and illumination variation was performed prior to analysis. Since the illuminator was a single light source and did not evenly cover the entire imaging surface, the center of the imaging plane was brighter than the outer edge from Figures 2(a)–2(c). The measured reflectance spectra consistently contained higher levels of noise at the two ends of the wavelength range as shown in Figure 2d. Thus, three steps were taken to mitigate these issues. First, a median filter length of 5 bands along the wavelength axis was applied to the reflectance spectra. Second, responses below 500 nm and above 900 nm were removed due to the high noise levels. Finally, we applied *l*_2_ normalization to reduce the magnitude variation caused by the point light source and focus more on the spectra shape [19, 25]. Specifically, we divided each spectral signature by its *l*_2_ norm [26]. The *l*_2_ normalization treats each spectrum as a vector and normalizes it to unit sphere in the vector space. The normalized result can be illustrated by comparing Figures 2(a), 2(d), and 2(e). In Figure 2(d), the blue curve, which is sampled from the brighter center in Figure 2(a), is greater than the red and orange curve. After normalization, the magnitude variation was reduced in Figure 2(e), and we can see that their spectra shape is consistent since they are sampled from the identical broccoli sample. Although the reflectance is extremely low in Figure 2(e), the signal and noise ratio (SNR) remains the same since the noise were reduced on the same scale.

After preprocessing the spectra, the regions of broccoli florets were segmented from the remainder of the hyperspectral cube. This segmentation was accomplished in two steps: (1) segmenting the broccoli sample from the black background and (2) segmenting the floret from the stalk. Spectral readings were analyzed from different regions of the image for step 1 (Figure 2(a)). There were clear spectral differences between broccoli and the black background (Figure 2(d) for raw spectra and Figure 2(e) for preprocessed spectra). Namely, the broccoli spectra (blue, red, and yellow spectra corresponding to colored points in Figure 2(a)) have a bump around 550 nm (visible bands of green) and a sharp increase around 700 nm (near infrared/red edge), whereas the spectra for the black background (purple, cyan, and green spectra corresponding to colored points in Figure 2(a)) are nearly flat up to 800 nm and then increase rapidly. We should note that after preprocessing steps, the differences in shape between broccoli and background are more distinguished in Figure 2(e). Given these significant spectral differences, the k-means clustering algorithms aim to iteratively partition the pixel spectra into two groups (i.e., broccoli and background in our case). Spectra were assigned to the group with the closest cluster centroid in Euclidean distance. The clustering result generated a binary mask image where the mask for a pixel would be 1 if its corresponding spectra belongs to the broccoli group; otherwise, the pixel mask would be 0. Next, a morphological image closing operation was applied on the mask image to connect any disconnected points. The segmented results are shown in Figure 2(b).

Since the glucosinolate concentration was measured on broccoli florets, we hypothesized that focusing on the spectra of the broccoli head was generated stronger correlation than analyzing the spectra of the entire broccoli (including the stem). In order to segment the head from the stalk, we applied the GraphCut algorithm [27] of the image segmentation toolbox in Matlab 2019b [28]. The algorithm was seeded by providing a marking that denoted the broccoli flower and the background including broccoli stem. The segmentation took around 5 to 10 seconds for each image. The segmentation results are shown in Figure 2(c).

The collected and preprocessed hyperspectral images consisted of 200 spectral bands of size 968 × 608 pixels. Each image usually covered around 40% to 80% of the broccoli sample and contained approximately 0.3 million of pixels per sample. Since the large data size will slow down the computing, the spectra were down-sampled to 5,000 per segmented broccoli sample via k-means clustering. Specifically, spectra of all the broccoli pixels were clustered into 5,000 groups, and each group spectra were represented by their average. Down-sampling was applied to the two segmentation scenarios being considered: (1) entire broccoli and (2) broccoli florets, respectively.

### 2.7. Spectral Unmixing with the SPICE Algorithm

The hyperspectral image is a high-dimensional image cube that described each pixel as the radiance or the reflectance at a range of wavelengths across the electromagnetic spectrum. The spectrum of a pixel is usually determined by the material of the object surface. These measured spectra are a mixture of a set of constituent spectrum, also known as endmembers. Spectral unmixing is the task defined as decomposing a mixed spectra into a collection of endmembers and their corresponding proportions, also known as abundances [29]. A well-known spectral model (and the most commonly applied to perform hyperspectral unmixing) is the linear mixing model (LMM), which represents each measured spectra as a convex combination of endmembers [29]:

(2) $$\begin{array}{c} {\text{s}}_{\text{i}}=\overset{\text{M}}{\underset{\text{k}=1}{\sum }}{\text{a}}_{\text{ik}}{\text{e}}_{\text{k}}+\varepsilon_{\text{i}}\text{such that }\overset{\text{M}}{\underset{\text{k}=1}{\sum }}{\text{a}}_{\text{ik}}=1,{\text{a}}_{\text{ik}}\in \left[0,1\right], \end{array}$$

where s_i_ is the spectra of pixel i, *ε*_i_ is the noise vector, *M* is the number of endmembers, e_k_ is the k^th^  endmember, and a_ik_ is the corresponding abundance value. The objective of unmixing is to estimate a set of endmembers and abundances that can indicate the freshness level of the broccoli being imaged. Particularly, the estimated endmembers are supposed to represent the range of “freshness” levels in the samples. Then, the associated abundances for the endmembers corresponding to “fresh” can be viewed as a freshness indicator. The endmembers and abundances were estimated using the Sparsity Promoting Iterated Constrained Endmember (SPICE) algorithm.

The SPICE algorithm benefited from simultaneous estimating the shape and number of endmembers as well as their abundances [22]. The Matlab and Python implementation for SPICE can be found here: github.com/GatorSense.

In our experiments, since the estimated endmembers highly depend on the parameter Γ (which serves as a parameter to determine the number of endmembers needed), a range of Γ values, starting from 10 to 150 in steps of 10, were explored. We conducted 10 repetitions of 3-fold cross-validation for 15 Γ values. With the estimated endmembers, the abundance feature can be derived from the training folds to fit the MLR model. Note that the large variation of errors is caused by the outliers in the training and validation folds. There was a greater prediction error on validation folds with a smaller Γ (Figures S3(a)-S3(b) and S3(f)-S3(g)), which indicates overfitting. In other words, since Γ determines the number of estimated endmembers to be eliminated, a smaller Γ value results in a greater number of endmembers and more parameters that need to be estimated (and provide opportunities for overfitting). There was a tendency of overfitting with an increasing number of endmembers, *M* (Figure S3(c)-S3(d) and S3(h)-S3(i)). In addition, both segmentation methods determined that *M* should be set at 3 (*M* = 3), since this value produced the most replications, over 450 (Figures S3(e) and S3(j)).

### 2.8. MI-ACE to Detect Freshness Indicator

In addition to spectral unmixing, we explored a target detection method as an alternative feature extraction approach. In particular, the Multiple Instance Adaptive Cosine Estimator (MI-ACE) [15, 16] was investigated to detect a spectral signature of “freshness.” In a hyperspectral image, the spectra s_i_ of an individual broccoli pixel i can be considered an instance, while all pixels of the entire broccoli sample (a group of instances) can be considered as a bag. A bag of one broccoli sample was labeled as positive if it contains high glucosinolates concentration, which meant that there existed at least one instance corresponding with the freshness indicator; otherwise, the bag was labeled as a negative. We set a threshold of glucosinolates level as 50 to distinguish less fresh (≥50) and fresh sample (<50). The threshold was decided by the significant increasing of glucosinolate level from day 1 to day 3.

MI-ACE estimates a discriminative target signature t for the freshness indicator. Then, instance s_i_ within one bag was assigned a confidence value a_i_, indicating the confidence of highly correlated with the discriminative target signature. Compared with unmixing approach, the MI-ACE in discriminative target signature was easier to interpret, implying that the freshness indicator can be distinguished from the negative instances of broccoli with low glucosinolates level. In this experiment, the target signatures are estimated in a similar setting of 3-fold cross-validation as SPICE.

### 2.9. Correlating Abundance with Glucosinolate Concentration Level

The estimated vectors $\hat{a}=\left\{{\hat{\text{a}}}_{1},{\hat{\text{a}}}_{2},\cdots ,{\hat{\text{a}}}_{\text{k}}\right\}\in {\mathbb{R}}^{1\times \text{M}}$ for each broccoli sample (where the value ${\hat{\text{a}}}_{\text{k}}$ is the average abundance for unmixing, or average confidence for MI-ACE of all pixels over the region of interest as ${\hat{\text{a}}}_{\text{k}}=1/\text{N}\sum_{\text{i}=1}^{\text{N}}{\text{a}}_{\text{ik}}$, where *N* denotes the number of pixels) were used to predict measured glucosinolate concentration values. Specifically, a multivariable linear regression (MLR) model (Equation (3)) was fit using least squares estimation approaches to predict the glucosinolate concentration value:

(3) $$\begin{array}{c} \text{glucosinolate}\sim \text{b}+\overset{\text{M}}{\underset{\text{k}=1}{\sum }}{\text{w}}_{\text{k}}{\hat{\text{a}}}_{\text{k}}, \end{array}$$

where *b* is the bias, *w*_*k*_ is the coefficient for ${\hat{\text{a}}}_{\text{k}}$, *M* is the number of estimated endmembers for unmixing, or *M* = 1 for MI-ACE.

### 2.10. PLSR Analysis

As a comparison approach, a PLSR model was investigated and compared with the above SPICE and MI-ACE approach. The average spectra of training samples were considered predictors matrix **X** with dimension (*N*_*training*_ × *B*_*bands*_), where *N*_*training*_ denotes the number of training samples and *B*_*bands*_ denotes the number of wavelength (*B*_*bands*_ = 200 in our case). The glucosinolate measurements were considered a response vector **Y** with dimension (*N*_*training*_ × 1). The partial least square is a latent variable approach to find the relations between predictors and response. The prediction of *Y* can be written as follows [30]:

(4) $$\begin{array}{c} \hat{Y}\sim T\beta , \end{array}$$

where ***T*** is the wavelength scores matrix and *β* is the regression coefficient. The regression was conducted using Matlab 2019b [28].

## 3. Results

### 3.1. Glucosinolate Content Increased during Postharvest Senescence

**Figure 3:**
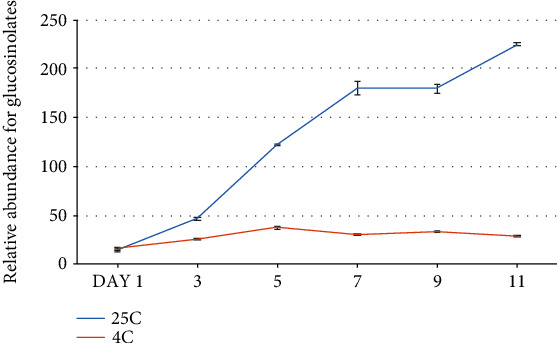
Quantification of the glucosinolates in broccoli by HPLC. The total glucosinolate level in broccoli florets sampled on days 1, 3, 5, 7, 9, and 11 of storage at room temperature (blue) or in the cold (red). The *y*-axis is the abundance of total glucosinolate content. Data represented means ± SE bars (*n* = 4 for each day).

When stored at room temperature, broccoli started to display visible yellowing after five days of harvest. There is no obvious color change found within a five-day storage. To assess the possibility that glucosinolate levels can be detected at the early stage of senescence and used as a indicator for senescence, we performed HPLC analysis to measure the total glucosinolate concentration during a twelve-day period in broccoli at either room (25°C) or cold (4°C) temperature. We found that there was a near-linear increase in glucosinolate concentration over the 12-day period when the broccoli was stored at room temperature (Figure 3). However, the accumulation of glucosinolates was not observed during cold storage. This data suggested that there was a strong correlation between glucosinolate levels and the progression of postharvest deterioration in broccoli when stored at room temperature.

### 3.2. Glucosinolate Biosynthesis Transcript Levels Increased during Postharvest Storage

**Figure 4:**
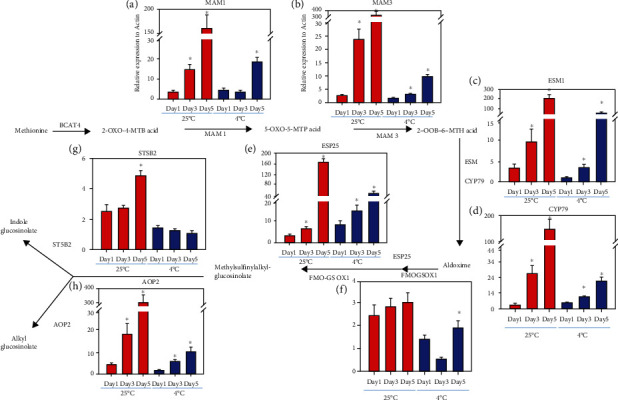
Transcript levels of genes in the glucosinolate biosynthetic pathway during room temperature and cold storage: *MAM1* (a), *MAM3* (b), *ESM1* (c), *CYP79* (d), *ESP25* (e), *FMO-GSOX1* (f), *ST582* (g), and *ADP2* (h). The transcript levels of each candidate gene are reported as the relative expression to Actin from samples stored at 25 °C (red) or 4 °C (blue) and sampled on days 1, 3, and 5. The genes encoding key enzymes are highlighted in yellow. The *y*-axis is the relative expression of each gene that was normalized using actin as an internal control. Data represents means ± SE bars (*n* = 3). The key enzymes were highlighted in yellow. Asterisks (∗) indicate statistically significant differences from day 1 (control) to day 3 or day 5 (storage temperature conditions) (*P* < 0.05).

To investigate whether the glucosinolate accumulation was due to glucosinolate biosynthesis and how the glucosinolate biosynthetic pathway was affected during postharvest senescence, we carried out quantitative gene expression analysis on the key genes in the glucosinolate biosynthetic pathway. The first reaction of glucosinolate biosynthesis was catalyzed by two enzymes methylthioalkylmalate synthases (MAM1 and MAM3). MAM1 and MAM3 initiated the formation of glucosinolate chain products. The transcripts of both enzymes that produce intermediates, like *MAM1* and *MAM3*, were increased (4.3-fold from day 1 to day 3 and 11-fold) from day 3 to day 5 at room temperature. In cold conditions, *MAM1* levels were undetectable on day 3 but increased on day 5 by 5.3-fold (Figure 4). This implied that *MAM1* levels were increased in higher proportions during room temperature storage. Similar patterns were observed for the other genes encoded enzymes such as *epithiospecifier modifier 1* (*ESM1*), *α*-ketoglutarate-dependent dioxygenase (*AOP2*), epithiospecifier protein (*ESP25*), *CYP79*, and *ST5*, as their transcripts increased significantly from day 0 to day 5. ESM1 and CYP79 modified and catalyzed the conversion of amino acid to aldoxime. However, under cold storage, the increase of transcript levels in flavinmonosygenases (FMOGSOX2) from day 0 to day 5 was not significant. The FMOGSOX2, an enzyme, catalyzed the conversion of methylthioalkyl glucosinolates to methylsulfinylalkyl glucosinolates. This observation provided evidence that changes in genes expression for the glucosinolate biosynthetic pathway were associated with the freshness of broccoli. Therefore, our results suggested that the production of glucosinolate was triggered by transcriptional regulation of glucosinolate biosynthesis during the postharvest storage. This data further validated that there is a correlation between postharvest senescence and glucosinolate production in broccoli when stored at room temperature, suggesting that the glucosinolate can be used to detect early senescence even without any visible color changes during storage.

### 3.3. Visualization of HSI Analysis

**Figure 5:**
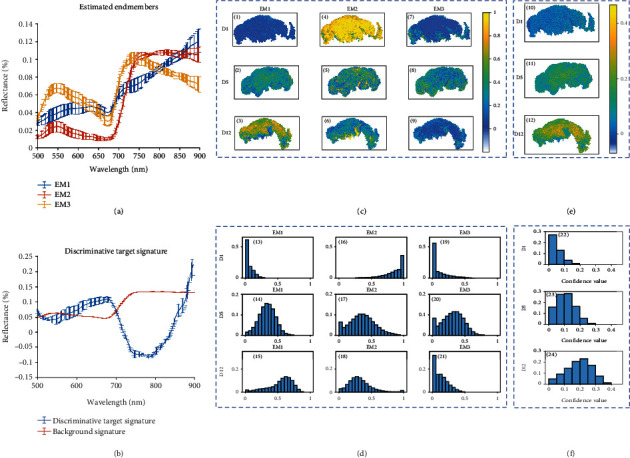
Visualization of HSI analysis of testing samples on days 1, 5, and 12, (a, c-d) for SPICE and (b, e-f) for MI-ACE. (a) Estimated endmembers using the SPICE methods on spectral images of broccoli florets. “EM” is an abbreviation of endmember. (b) Estimated discriminative target and background signature using the MIACE methods. (c) Abundance map of estimated endmembers for testing samples on day 1, day 5, and day 12. (d) Histogram of abundance value. The legend of *x*-axis and *y*-axis are the abundance value and their proportion, respectively. (e) Confidence map of target detected by MI-ACE for testing samples on day 1, day 5, and day 12. (f) Histogram of confidence value. The legend of *x*-axis and *y*-axis are the confidence value and their proportion, respectively.

To validate if HSI imaging can detect the glucosinolate contents in broccoli, we performed imaging analysis on broccoli at two storage conditions after harvest and designed two approaches to test out hypothesis. In Figure 5, it showed the HSI analysis on broccoli florets with two approaches, the SPICE (Figures 5(a) and 5(c)–5(d)) and the MI-ACE (Figures 5(b) and 5(e)–5(f)) algorithm.

First, the estimated endmembers via SPICE are shown in Figure 5(a). “EM” is an abbreviation of endmember. The error bar in Figure 5(a) illustrated the variance of estimated endmembers over 10 replications of 3-fold cross-validation as stated in Materials and Methods. It was noted that the SPICE algorithm was unsupervised. The information about the estimated endmembers was inferred by examining the abundance map. Figure 5(c) compared the abundance map for the testing sample. The rows were grouped by time course (1, 4, and 7 for day 1; 2, 5, and 8 for day 5; 3, 6, and 9 for day 12), while each column was corresponding to an estimated endmember (1-3 for EM1, 4-6 for EM2, 7-9 for EM3). The numeric values in abundance map were mapped into the color scale, where the blue denotes weak abundance near the 0, and bright yellow denotes strong abundance near the 1. Figure 5(d) shows the histograms of abundance maps in the same layout (rows for time course and columns for estimated endmembers). For instance, Figure 5(d) (13) was the histogram of abundance values showing in Figure 5(c)(1), indicating how much the broccoli sample is correlated to EM1 in day 1.

Abundance maps and histograms shown in Figures 5(c) and 5(d) are informative to classify estimated endmember via SPICE. For example, the great abundance value (bright yellow) in Figure 5(c) (2) and dense concentration of histogram to 1 in Figure 5(d) (16) implied that the broccoli sample from day 1 was highly correlated with EM2 (shown in Figure 5(a)). Thus, EM2 is potentially the “fresh endmember.” Similarly, Figure 5(c) (3) and Figure 5(d) (15) implied that the broccoli sample from day 12 had a strong response to EM1 (shown in Figure 5(a)). Thus, EM1 is potentially the “least fresh endmember.” Taken together, the distribution of abundance values were associated with each endmember that provided information of freshness over the time course.

The visualization of the MI-ACE result is shown in Figures 5(b), 5(e), and 5(f), in which (b) plotted the discriminative target (less fresh) and background (fresh) signature on broccoli florets. The background signature was estimated by averaging the negative bag, which means the spectra of fresh broccoli with low glucosinolate level. The discriminative signature was estimated via MI-ACE algorithm and implied the difference between fresh and less fresh broccoli. Similar to Figure 5(a), the error bar in Figure 5(b) illustrated the variance of estimated signatures over 10 replications of 3-fold cross-validation. According to the Figure 5(b), the increasing of glucosinolates resulted in several changes of broccoli spectra, including a bump (increasing) around 580-700 nm (visible bands of yellow, orange, and red) and a concave (decreasing) around 750-800 nm (near infrared). The shape of discriminative target signature was consistent with the estimated endmembers in Figure 5(a), where the reflectance of the EM1 (least fresh) was greater around 580-700 nm and less around 700-800 nm compared with EM2 (most fresh). Based on the discussion, 580-700 nm and 750-800 nm are potential for accounting freshness of broccoli. It is also worth noting that, by examining the variance in Figure 5(b), the discriminative signature has a greater variance between 550 and 700 nm, which can be explained by the variance on visual appearance, while the variance between 700 and 900 nm is relatively smaller, which means the detected signature are more consistent in the near-infrared wavelengths. Our future work will include investigating the most significant wavelength via band selection techniques.

Figures 5(e) and 5(f) show the confidence map and their histograms for testing samples on day 1, day 5, and day 12. The confidence values implied the score that each pixel belongs to the target (less fresh). It shows in Figure 5(e) that the confidence was increasing from day 1 (11) to day 12 (12) and the concentration of corresponding histograms were shifted from 0 in day 1 (22) to 1 in day 12 (24). This change implied the tendency of getting less fresh over time course. Therefore, the distribution of confidence values was correlated with freshness over the time course, which was also consistent with Figures 5(c)–5(d).

### 3.4. Correlating the Changes in Glucosinolate Levels in Postharvest Broccoli through Hyperspectral Imaging

**Figure 6:**
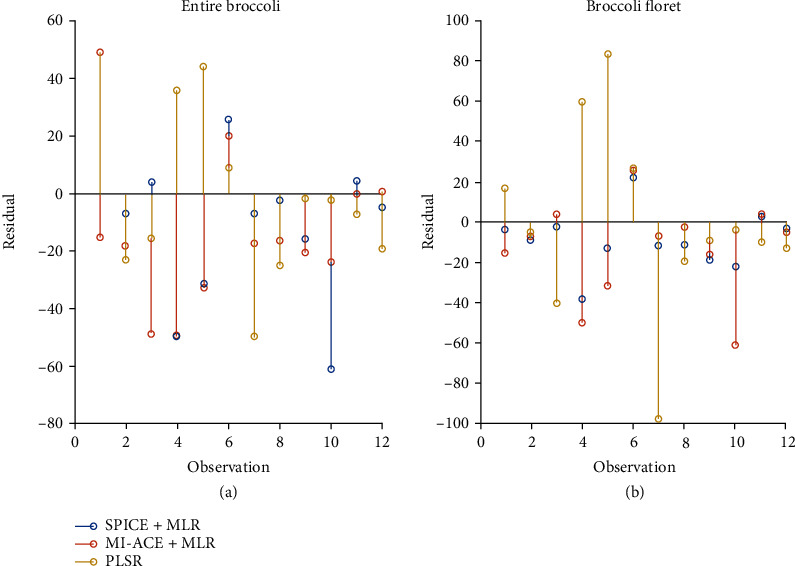
Residuals of predicted glucosinolate levels on testing fold. (a) Residuals on the entire broccoli. (b) Residuals on the broccoli florets. The *x*-axis indicates the observations. The *y*-axis indicates the residuals that subtracted the predicted values from the measured values. Markers in various colors denote the glucosinolate levels predicted by the different methods. Markers that are closer to 0 are more accurate predictions.

To correlate the hyperspectral image with the glucosinolate levels in postharvest broccoli, we investigated both the abundance feature generated via SPICE, and the confidence value generated via MI-ACE. The glucosinolate concentration and derived abundance feature as well as confidence value were applied to fit the (MLR) model. In addition, we compared the feature extraction + MLR model approaches with the classical PLSR model. 6 fitted models with variables based on different approaches were applied to the testing sample to generate the residuals shown in Figure 6. The residuals are calculated by $Res=Y-\hat{Y}$, where **Y** denotes the measured glucosinolates concentration and $\hat{Y}$ denotes the predicted value. It can be seen from Figure 6 that SPICE+MLR generated less residuals in most of observations.

In addition, the F-test of overall significance was conducted to evaluate the multivariable linear regression models and the PLSR model. F statistics and the corresponding *P* values are shown in Table 2. All the *P* values were less than the significant level of 0.1%, indicating that the derived models fit significantly better than a degenerate model with no predictor variables.

### 3.5. Result Verification

The above models were first trained by cross-validation and then evaluated on the testing set to verify its effectiveness on validation and testing set. The entire datasets were split into training, validation, and testing folds. To be more specific, 48 samples under 12 conditions (2 storage conditions over 6 time points, each condition including 4 replicates) were randomly divided into 4 groups, one for testing and the other 3 for training and validation. Each fold contained 12 samples; 1 replication randomly selected from each condition. The training and validation dataset were shuffled in every repetition.

The training process was conducted for 10 repetitions over 3-fold. In each repetition, we trained the model by 3-fold cross-validation and tested the trained model on the testing fold, by calculating the mean and standard deviation of the testing and training prediction error (Table 3). The results shown in Figure 6 are generated by the model that was selected according to the root means square error (RMSE) and *R*-squared value from training and validation folds. The primary observation from Table 3 is that the SPICE+MLR and MI-ACE+MLR approaches outperformed the PLSR model on the testing error, implying less overfitting. Note that the better performance of the testing data (as compared to the training set) is explained in the discussion about sample outliers below.

### 3.6. Outliers

The smaller value of the prediction error for the testing fold compared with training and validation folds in Table 2 can be explained by the observed outliers (Figure S1). The prediction performance of the training fold using the SPICE method is plotted against the ground-truth, with the marker size and color related to their prediction error. Apparently, the three circled outliers in each generated a greater amount of prediction error compared to the other data points. Table S1 lists the glucosinolate concentrations of 4 replications stored in room temperature at each of the 6 time points. The three bold numbers correspond to the circled outliers in Figure S1. In replicate 1, the glucosinolate concentration was increased along days, while in replicates 2-4, the bold numbers showed “abnormal” performance. An additional experiment was conducted, where the outliers were moved to the testing fold. Figure S2 and Table S2 show the prediction performance and error of this additional testing fold.

## 4. Discussion

Senescence is an important and fleeting state, and its onset and progression after harvest cannot be easily detected. The current lack of objective indices for defining tissue senescence in fruit and vegetables limited our capacity to control product quality and leads to food waste. However, senescence is a physiological process initiated at or near harvest that can be tracked by monitoring well-described changes in gene expression, physiological process, and metabolomic signatures. In this work, we propose that glucosinolates are associated with discrete stages of senescence, for potential use as diagnostic indicators of freshness. Using HPLC and quantitative real-time PCR analysis, we evaluated glucosinolate concentration and the expression of key genes in glucosinolate biosynthetic pathway in postharvest broccoli. We found that there is a linear correlation between the glucosinolate production and postharvest senescence in broccoli. This lineage of glucosinolate could result from the metabolic accumulation of glucosinolates during progression of senescence in stored broccoli. Our data determined that transcriptional level of glucosinolate biosynthesis increased rapidly when stored at room temperature. Therefore, we demonstrated that glucosinolate content can be used to detect the early senescence potentially serving as a “freshness indicator” in broccoli and to define a freshness signature when stored at room temperature.

We further developed the imaging analysis on the initiation and progression of senescence in broccoli. We performed two featured extraction approaches in estimating the endmembers (a.k.a. signatures) of broccoli tissue over storage period. The estimated abundance value via SPICE and confidence value via MI-ACE approach not only visualized the broccoli in different postharvest stage, but also indicated the changes in glucosinolate concentration values. The prediction error was explained by the fact that the measurements of the hyperspectral data and the glucosinolate concentrations were conducted across different sample scales. Namely, the abundance values are derived from imaging across the entire surface of one side of a broccoli sample, whereas the glucosinolate value was measured using only one small component of the broccoli tissue. The RMSE values showed that unmixing the broccoli florets only has slightly less error than using the entire broccoli sample. Although the RMSE values of MI-ACE did not show much improvement compared with unmixing methods, the estimated discriminative target signature was easier to interpret, implying the specific wavelength where the broccoli spectra were affected by increasing glucosinolates concentrates. In addition, we compared the prediction result with the PLSR approach and showed that our feature extraction + MLR model were more stable and less overfitting.

In summary, hyperspectral imaging held promising strength in demonstrating state-of-the-art performance in crop sciences through the modulation of imaging with spectroscopy. As shown in this effort, HSI had the potential to provide quantitative parameters in detecting the content of glucosinolates that associated with postharvest senescence. The accumulation of glucosinolates marks the beginning of senescence before it is visible in postharvest broccoli. Our results can be directly extended to the other fresh crucifers such as cabbage, kale, and cauliflowers as well as the other fresh produce. The outcomes of the results will provide insights into early detection of deterioriation of fruit and vegetables throughout the food production pipeline, therefore understand how food waste can be reduced.

## Figures and Tables

**Table 1:** List of primers for quantitative PCR performed for glucosinolate biosynthesis pathway in broccoli.

Glucosinolate biosynthetic genes	Sequence
BO_ACTIN2-FORWARD	TGGTCGTGACCTTACTGACTAT
BO_ACTIN2-REVERSE	TCACTTGTCCGTCGGGTAAT
BO_ST5B2-FORWARD	CCCATATACCCAACGGGTCG
BO_ST5B2-REVERSE	CCCATGAACTCAGCCAACCT
BO_MAM1-FORWARD	GGAATTATCCCTACCACCAGTTC
BO_MAM1-REVERSE	CAGAGGAGCAACATGAGATGAG
BO_CYP79F1-FORWARD	GTTAGGACAAGCGGAGAAAGA
BO_CYP79F1-REVERSE	CCATCAATGTTCCAACCTCTAAAC
BO_AOP2-FORWARD	GTGAGGAGTGATGTCCGTAAAG
BO_AOP2-REVERSE	GCCTCAACTGGTAACTCGAAA
BO_ESM1-FORWARD	CCGGAAGTAGCGTTGTTTACT
BO_ESM1-REVERSE	GTTAGGGTCGTCAAGGGATTT
BO_MAM3-FORWARD	ATCGTCCGTACAACAAGTCATC
BO_MAM3-REVERSE	GTATGTACTCTGGCCACCTTTC
BO_ESP-FORWARD	AGGACGATCGAGGCCTATAA
BO_ESP-REVERSE	GAATCCAGCTCCACCTCTTT
BO_FMOGSOX1-FORWARD	GGATTAATAGCGGCCAGAGAG
BO_FMOGSOX1-REVERSE	GCGGGTCGGATTCAGATTTA

**Table 2:** Significant test for multivariable linear regression model.

	SPICE+MLR		MI-ACE+MLR		PLSR	
	F statistics	*P* value	F statistics	*P* value	F statistics	*P*value
Entire broccoli	23.12	4.93e-6	124.96	1.53e-10	15.42	4.28e-8
Broccoli florets	51.24	8.34e-9	115.35	3.24e-10	15.48	4.11e-8

**Table 3:** The error of prediction of glucosinolate levels using MLR model with abundances calculated by SPICE, confidence calculated by MI-ACE, as well as the PLSR model.

		SPICE + MLR		MI-ACE + MLR		PLSR	
		RMSE	*R* ^2^	RMSE	*R* ^2^	RMSE	*R* ^2^
Entire Broccoli	Training and validation	44.93 ± 1.37	0.72 ± 0.02	46.14 ± 3.12	0.71 ± 0.04	42.94 ± 1.09	0.74 ± 0.01
	Testing	26.14 ± 4.87	0.85 ± 0.06	30.21 ± 9.70	0.81 ± 0.11	31.00 ± 2.61	0.80 ± 0.03

Broccoli Florets	Training and validation	44.78 ± 1.23	0.72 ± 0.02	47.94 ± 1.93	0.68 ± 0.03	45.97 ± 8.74	0.70 ± 0.12
	Testing	21.34 ± 3.26	0.90 ± 0.03	23.67 ± 2.99	0.88 ± 0.03	36.69 ± 7.92	0.70 ± 0.12

## Data Availability

The data used to support the findings of this study are included within the article.

## Additional Points

No seed has been collected. The plant materials were harvested from the local farm followed by the permissions and licenses of Florida agricultural guideline. The experimental research on plants was complied with institutional, national, and international guidelines and legislation.

## Supplementary Materials

Supplementary Materials Figure S1: predicted glucosinolate levels in training folds. The *x*-axis indicated the real glucosinolate concentration, and the *y*-axis indicated the predicted values. Markers that are closer to the “*x = y*” line are more accurate predictions. The marker size and color corresponded to the prediction error; the bigger and brighter markers indicated greater error. Figure S2: residuals of predicted glucosinolate levels on additional testing fold. (a) Residuals on the entire broccoli. (b) Residuals on the broccoli florets. The *x*-axis indicates the observations. The *y*-axis indicates the residuals that subtracted the predicted values from the measured values. Markers in various colors denote the glucosinolate levels predicted by the different methods. Markers that are closer to 0 are more accurate predictions. Figure S3: exploration of SPICE parameters. (a-e) The training and validation errors across various parameter settings for the entire broccoli, in replicate. (f-j) The training and validation errors across various parameter settings for broccoli florets. Specifically, (a) and (f) measure the RMSE over *Γ*, (b) and (g) measure the R2 over *Γ*, (c) and (h) measure the RMSE over M, (d) and (i) measure the R2 over M, and (e) and (j) show the histogram of M over all replications. Table S1: the glucosinolate concentration under 25 °C on each sampling day. Table S2: comparison of prediction error on additional testing fold.
