# Supplementary material for: Evaluation of Postharvest Senescence of Broccoli via Hyperspectral Imaging
Source: Plant Phenomics. 2022 May 9;2022:9761095. doi: 10.34133/2022/9761095 (PMC9115666; doi:10.34133/2022/9761095)
Supplement: Supplementary Materials — Figure S1: predicted glucosinolate levels in training folds. The x-axis indicated the real glucosinolate concentration, and the y-axis indicated the predicted values. Markers that are closer to the “x = y” line are more accurate predictions. The marker size and color corresponded to the prediction error; the bigger and brighter markers indicated greater error. Figure S2: residuals of predicted glucosinolate levels on additional testing fold. (a) Residuals on the entire broccoli. (b) Residuals on the broccoli florets. The x-axis indicates the observations. The y-axis indicates the residuals that subtracted the predicted values from the measured values. Markers in various colors denote the glucosinolate levels predicted by the different methods. Markers that are closer to 0 are more accurate predictions. Figure S3: exploration of SPICE parameters. (a-e) The training and validation errors across various parameter settings for the entire broccoli, in replicate. (f-j) The training and validation errors across various parameter settings for broccoli florets. Specifically, (a) and (f) measure the RMSE over Γ, (b) and (g) measure the R2 over Γ, (c) and (h) measure the RMSE over M, (d) and (i) measure the R2 over M, and (e) and (j) show the histogram of M over all replications. Table S1: the glucosinolate concentration under 25 °C on each sampling day. Table S2: comparison of prediction error on additional testing fold. [file 9761095.f1.docx]

**Supplementary information**

**Fig. S1 Predicted glucosinolate levels in training folds.** x-axis indicated the real glucosinolate concentration, y-axis indicated the predicted values. Markers that are closer to the “x = y” line are more accurate predictions. The marker size and color corresponded to the prediction error, the bigger and brighter markers indicated greater error.


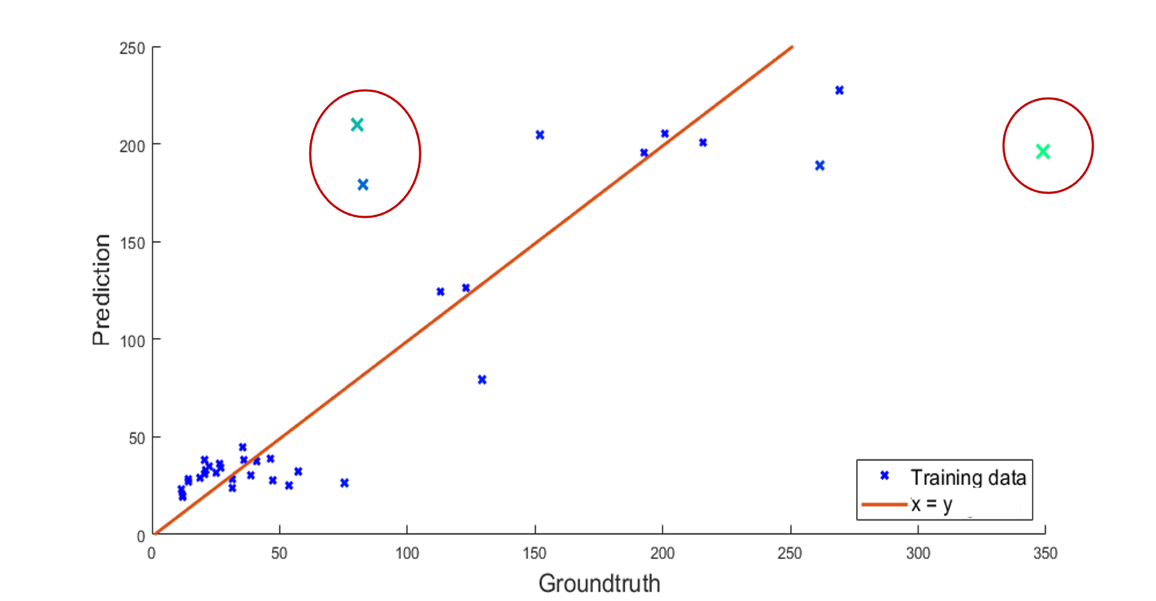


**Fig. S2 Residuals of predicted glucosinolate levels on additional testing fold.** (a) Residuals on the entire broccoli. (b) Residuals on the broccoli florets. x-axis indicates the observations. The y-axis indicates the residuals that subtracted the predicted values from the measured values. Markers in various color denote the glucosinolate levels predicted by the different methods. Markers that are closer to 0 are more accurate predictions.


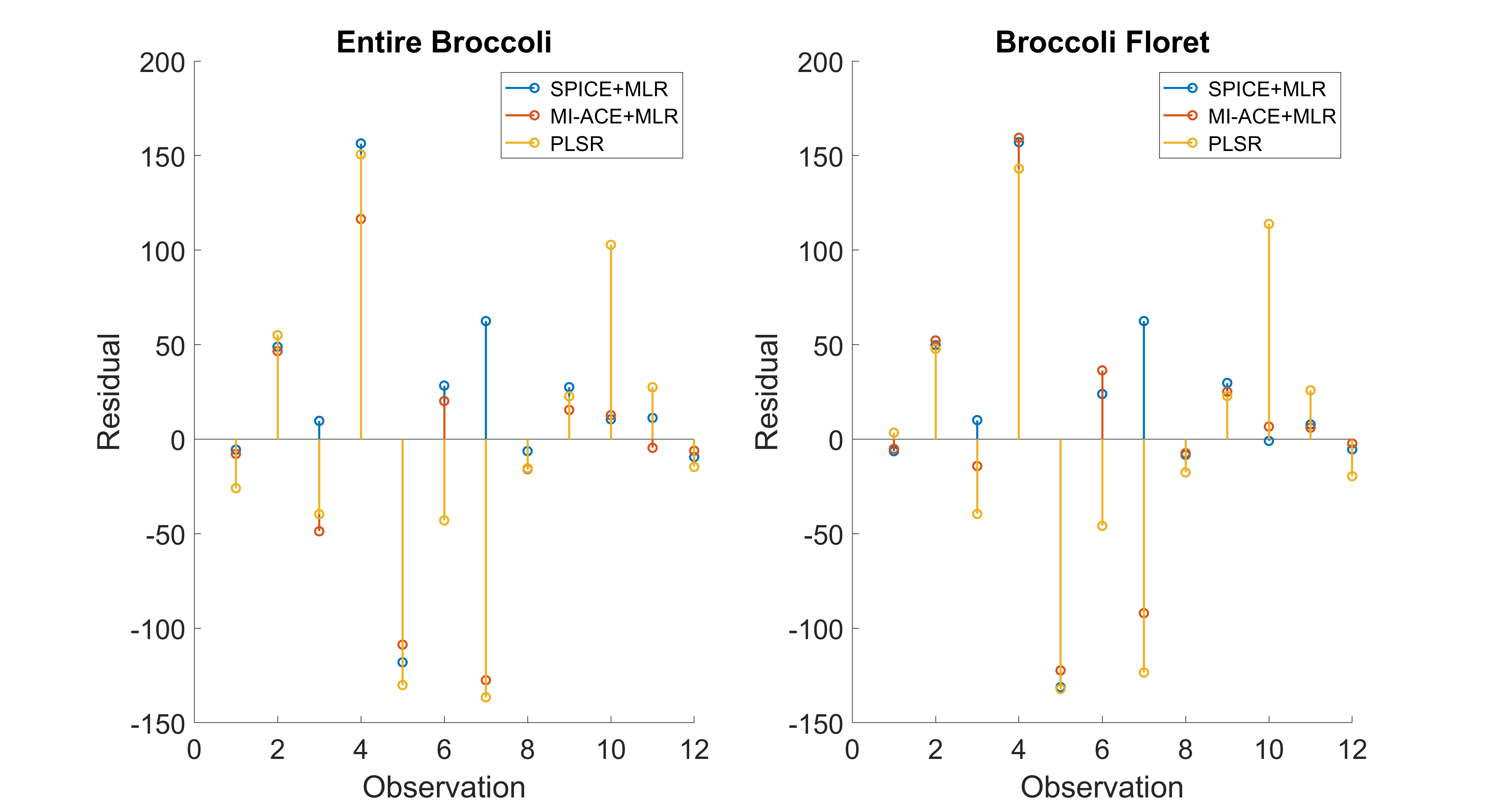


**Fig. S3 Exploration of SPICE parameters**

(a-e) The training and validation errors across various parameter settings for the entire broccoli, in replicate. (f-j) The training and validation errors across various parameter settings for broccoli florets. Specifically, (a) and (f) measure the RMSE over $\Gamma$, (b) and (g) measure the R^2^ over $\Gamma$, (c) and (h) measure the RMSE over *M*, (d) and (i) measure the R^2^ over *M*, (e) and (j) show the histogram of *M* over all replications.


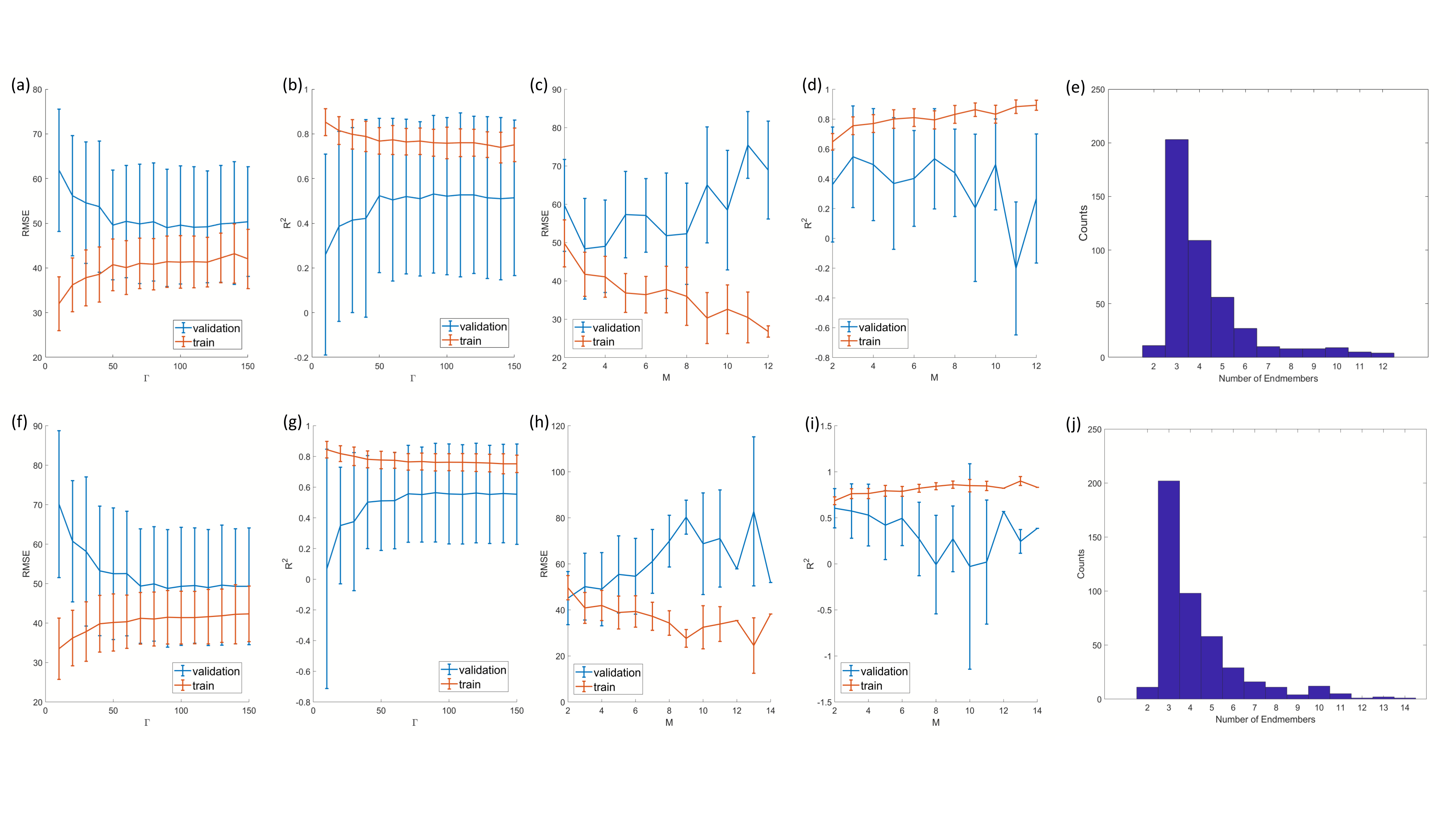


**Table S1. The glucosinolate concentration under 25 °C on each sampling day**

|  | **Rep 1** | **Rep 2** | **Rep 3** | **Rep 4** |
| --- | --- | --- | --- | --- |
| **Day1** | 11.9544 | 11.9405 | 14.2901 | 21.099 |
| **Day3** | 53.858 | 31.6877 | 75.4149 | 25.0054 |
| **Day5** | 129.0532 | 113.1505 | 122.6086 | 122.941 |
| **Day8** | 135.2934 | 151.8804 | **348.884** | **82.8482** |
| **Day10** | 182.7224 | **80.5022** | 192.528 | 261.347 |
| **Day12** | 200.8352 | 269.094 | 215.7358 | 213.3986 |

**Table S2. Comparison of prediction error on additional testing fold**

|  | | **SPICE+MLR** | | **MIACE+MLR** | | **PLSR** | |
| --- | --- | --- | --- | --- | --- | --- | --- |
|  |  | **RMSE** | **R^2^** | **RMSE** | **R^2^** | **RMSE** | **R^2^** |
| **Entire**  **Broccoli** | **Training &Validation** | 29.24 ± 1.89 | 0.85 ± 0.02 | 36.69 ±3.13 | 0.85 ± 0.04 | 32.17 ±3.13 | 0.82 ± 0.08 |
|  | **Testing** | 61.81 ± 2.71 | 0.58 ± 0.05 | 63.56 ± 5.43 | 0.53± 0.08 | 69.53 ± 9.59 | 0.43± 0.16 |
| **Broccoli**  **florets** | **Training &Validation** | 27.54 ± 0.42 | 0.86 ± 0.01 | 39.07 ± 1.16 | 0.87 ± 0.04 | 31.48 ± 8.64 | 0.82 ± 0.09 |
|  | **Testing** | 62.47 ± 0.70 | 0.58 ± 0.01 | 67.67 ± 1.28 | 0.47 ± 0.03 | 71.30 ± 6.45 | 0.41 ± 0.11 |
